# Development of a salmon-derived crosslinked atelocollagen sponge disc containing osteogenic protein-1 for articular cartilage regeneration: *in vivo* evaluations with rabbits

**DOI:** 10.1186/1471-2474-14-174

**Published:** 2013-05-30

**Authors:** Hiroyuki Mori, Eiji Kondo, Yasuyuki Kawaguchi, Nobuto Kitamura, Nobuhiro Nagai, Hirokazu Iida, Kazunori Yasuda

**Affiliations:** 1 Department of Sports Medicine and Joint Surgery, Hokkaido University Graduate School of Medicine, Kita-15, Nishi-7, Kita-ku, Sapporo 060-8638, Japan; 2 Department of Orthopaedic Surgery, Kansai Medical University, Osaka, Japan; 3 Department of Translational Research, Tohoku University School of Medicine, Sendai, Japan

**Keywords:** Salmon-derived Atelocollagen Sponge, Crosslinked Collagen, Cartilage Regeneration, Scaffold, Biomaterial

## Abstract

**Background:**

We have developed crosslinked salmon-derived atelocollagen sponge, which has a denaturation temperature of 47 degrees Celsius. The purpose of this study is to evaluate the fundamental in vivo efficacy of the osteogenic protein (OP) -1 containing salmon-derived collagen sponge disc (SCS) on cartilage regeneration, using a rabbit model.

**Methods:**

A total of 24 rabbits were used in this study. In each animal, a full-thickness osteochondral defect was created in each femoral trochlea. Then, each 12 rabbits were randomly divided into the two groups. In Group I, an OP1-SCS disc was implanted into the defect in the right knee. In Group II, a SCS disc without OP-1 was implanted into the defect in the right knee. A control group of 12 rabbits was assembled from randomly-selected left knees from among the first two groups. In Group-III, we applied no treatment for a defect in the left knee to obtain the untreated control. All rabbits were sacrificed at 12 weeks after surgery. In each group, 10 animals were used for histological and immunohistological evaluations, and the remaining 2 were used for real-time polymerase chain reaction (PCR) analyses.

**Results:**

In Group I, a regenerated cartilage tissue rich in proteoglycan and type-2 collagen was found at 12 weeks, although the width was thicker than that of Group II. In Group II, the defect was filled with thick inhomogeneous tissues, including cartilage, fibrous, and bone tissues at 12 weeks. Concerning the gross observation and histological scores at 12 weeks, the ANOVA showed significant differences (p < 0.0001, and p < 0.0001, respectively). The post-hoc test indicated that the gross observation and histological scores of Group I was significantly greater than those of Groups II (p = 0.035, and p = 0.0104, respectively) and III (p < 0.0001, and p < 0.0001, respectively), while Group II was significantly greater than Group III (p = 0.0069, and p = 0.005, respectively). The real time PCR analysis showed that gene expression of type-2 collagen and aggrecan of Group I was greater than that of Group II.

**Conclusions:**

The present study clearly demonstrated that the implantation of the OP1-SCS disc without any cultured cells may induce spontaneous hyaline-like cartilage regeneration to greater degrees than implantation of only the salmon-derived collagen sponge disc.

## Background

Functional repair of an articular osteochondral defect remains a major challenge in orthopaedic surgery [1-4]. Bovine and porcine atelocollagen materials have commonly been used as useful scaffolds. Mammalian-derived atelocollagens, however, carry potential risks of disease (zoonosis) transmission, such as bovine spongiform encephalopathy (BSE), foot-and-mouth disease, and so on, to humans [5]. On the other hand, fish-derived atelocollagen is known to be safe for human beings concerning the zoonosis transmission [6]. In addition, fish atelocollagen has a potentially large pool source with a low cost [5]. Therefore, fish atelocollagen has a potential that can be an alternative to animal collagen. Fish-derived atelocollagen, however, has not yet been used as a medical biomaterial because of its low denaturation temperature [7]. Namely, fish-derived atelocollagen is transformed into gelatin at a human body temperature.

**Figure 1:**
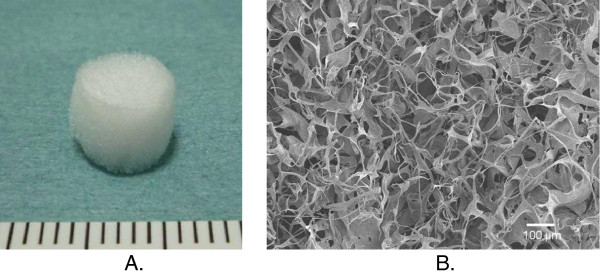
A salmon-derived cross-linked atelocollagen sponge disc containing OP-1 (OP-1SCS disc) as a novel cell-free device for cartilage repair (A) and the ultrastructure of the atelocollagen sponge (B).

Recently, however, we have developed a cross-linked salmon-derived atelocollagen, which is not transformed into gelatin even at a human body temperature [8,9]. Our recent *in vitro* studies have shown that the cross-linked salmon-derived atelocollagen has a better ability concerning cell proliferation in the surface culture than commonly used swine-derived atelocollagen [10,11]. Therefore, we have studied to develop a cell-free device for cartilage repair, which can be implanted without cell culture into an osteochondral defect. In our preliminary *in vivo* study using rabbits, implantation of the salmon-derived cross-linked atelocollagen into an osteochondral defect induced hyaline-like cartilage regeneration in the defect, while the commonly available porcine collagen does not have such effects [12]. However, the inductive ability of hyaline-like cartilage regeneration is not sufficient because the width of the regenerated cartilage tissue was approximately a half of the normal articular cartilage width. A possible solution to this issue is to create a composite collagen material containing some signaling molecules, which can enhance the cartilage regeneration ability of salmon-derived cross-linked atelocollagen. We have focused on osteogenic protein (OP)-1, which is also called bone morphogenetic protein-7 (BMP-7). OP-1 is known as a potent signaling molecule, which has been clinically applied for bone and cartilage repair [13], because they are produced endogenously by human adult articular chondrocytes and are able to stimulate the synthesis of cartilage extracellular matrix components [14-16]. In addition, OP-1 is shown to exhibit not only pro-anabolic but also anticatabolic activities for cartilageous tissues [17]. Thus, we have developed a salmon-derived cross-linked atelocollagen sponge disc containing OP-1 as a novel cell-free device for cartilage repair (Figure 1).

The purpose of this study is to evaluate the fundamental *in vivo* efficacy of the OP-1 containing salmon-derived collagen sponge disc (abbreviated as “OP1-SCS disc”) on cartilage regeneration, using a rabbit model. Specific hypotheses in this study include, first, that the OP1-SCS disc may not show any detrimental effects in the knee joint, when they were implanted into an osteochondral defect created in the rabbit knee. The second hypothesis is that the implanted OP1-SCS disc may be completely absorbed at 12 weeks. The third hypothesis is that the implantation of the OP1-SCS disc without any cultured cells may induce spontaneous hyaline-like cartilage regeneration to greater degrees than implantation of only the salmon-derived collagen sponge disc.

## Methods

### Preparation of the salmon-derived collagen sponge disc containing OP-1

The crosslinked atelocollagen sponge disc was prepared from the fresh skin of chum salmon by acid solubilization and subsequent pepsin digestion, according to a procedure previously reported [6]. Briefly, the lyophilized salmon-derived collagen was added to 100 mL of diluted HCl (pH 3) to a concentration of 0.5% (w/v) and stirred overnight at 4 degrees Celsius. Next, 1 ml of the collagen solution was transferred into a multiple 48-well plate for tissue culture (Asahi Techno Glass, Tokyo, Japan) and then frozen at −70 degrees Celsius for 12 hours. The frozen plates were placed into a lyophilizer (FDU-830, Tokyo Rikakikai, Tokyo, Japan) for 24 hours. The obtained porous materials were immersed in a 4-M NaCl aqueous solution containing 1% (w/v) water-soluble EDC (Dojindo, Tokyo, Japan) and kept at 4 degrees Celsius for 24 hours. These materials were washed three times with diluted water and then lyophilized again. Thus, we obtained the crosslinked atelocollagen sponge. The degree of crosslinking (the decrease ratio in the free amine group contents of the crosslinked sample to the uncrosslinked sample) of this collagen sponge was approximately 40% [12]. Our previous in vivo study [12] showed that the crosslinked SCS sponge did not show any detrimental effects in the knee joint, when they were implanted into an osteochondral defect created in the rabbit knee. From this material, we created the OP1-SCS disc for implantation in the present study (Figure 1). Each disc had a diameter of 4.5 mm and a height of 3 mm. We injected 10-μg OP-1 (R&D Systems, Minneapolis, MN, USA) dissolved in 1-ml saline solution into a core portion of the disc immediately before implantation. This dose of OP-1 was chosen based on a previous study [18].

### Study design and animal experimentation

Animal experiments were carried out in the Institute of Animal Experimentation, Hokkaido University School of Medicine under the Rules and Regulation of the Animal Care and Use Committee. A total of 24 mature female Japanese white rabbits, weighing 3.9 ± 0.5 kg, were used in this study. An operation for each animal was performed under intravenous anesthesia (pentobarbital, 25 mg/kg) under sterile conditions. In each animal, the bilateral knee joints were exposed through a medial parapatellar incision, and the patella was dislocated laterally. A full-thickness osteochondral defect having a diameter of 4.3 mm and a depth of 3 mm was created in each femoral trochlea, using a sharp drill. Then, in 12 of the 24 rabbits, we implanted an OP1-SCS disc into the defect in the right knee (Group-I knees) using the press-fit technique. In the remaining 12 rabbits, we implanted a salmon-derived collagen sponge disc without OP-1 into the defect in the right knee (Group-II knees) in the same manner. In each group, we applied no treatment for a defect in the left knee to obtain the untreated control (Group-III knees). The incised joint capsule and the skin wound were closed in layers with 3–0 nylon sutures, and an antiseptic spray dressing was applied. Postoperatively, each animal was allowed unrestricted activity in a cage (310 × 550 × 320 mm) without any joint immobilization. All rabbits were sacrificed at 12 weeks after surgery. This period was chosen because it was considered to be the best period to compare the degree of cartilage regeneration among the 3 groups according to the previous study [12]. The knee joints were immediately removed en bloc with the surrounding tissue. In each group, 10 animals were used for histological and immunohistological evaluations, and the remaining 2 were used for real-time polymerase chain reaction (PCR) analyses.

### Gross observations and histological evaluations

At the time of sacrifice, the tissue regenerated in the osteochondral defect underwent gross observations, and was quantitatively evaluated with the grading scale reported by Wayne et al. [19]. The maximum total score was 16 points. A distal portion of the resected femur was fixed in a 10% neutral buffered formalin solution for 3 days, decalcified with 50 mM ethylenediaminetetraacetic acid for a period of 3–4 weeks, and then cast in a paraffin block. The femur was sectioned perpendicular to the longitudinal axis, and stained with Hematoxylin-Eosin and Safranin-O. For immuno-histological evaluations, monoclonal antibody (anti-hCL(II), purified IgG, Fuji Chemical Industries Ltd, Toyama, Japan) was used as primary antibodies. Immunostaining was carried out according to the manufacturer’s instructions using the Envision immunostaining system (DAKO Japan, Kyoto, Japan). Finally, the sections were counterstained with hematoxylin. Histology was evaluated with a scoring system reported by Wayne et al. [19]. Subsequently, the maximum total score was 19 points. In addition, for each defect, randomly selected 3 sections of central portion with Safranin-O were evaluated by 2 authors (E.K., Y.K.) under a blinded manner. Cellularity of the nuclei in the proteoglycan-rich tissue were observed with light microscopy.

### Real-time PCR analysis

Total RNA was extracted from the tissues regenerated in the defect using the RNeasy mini kit (Qiagen Inc., Valencia, CA) at 12 weeks. RNA quality from each sample was assured by the A260/280 absorbance ratio. The RNA (100 ng) was reverse-transcribed into single strand cDNA using PrimeScript® RT reagent Kit (TakaraBio, Ohtsu, Japan). The RT reaction was carried out for 15 min at 37 degrees Celsius and then for 5 sec at 85 degrees Celsius. All oligonucleotide primer sets were designed based upon the published mRNA sequence. The expected amplicon lengths ranged from 93 to 189 bp. The sequences of primers used in real time PCR analyses for rabbit regenerative tissues were as follows: type 2 collagen forward GACCATCAATGGCGGCTTC; reverse CACGCTGTTCTTGCAGTGGTAG. Aggrecan forward GCTACGACGCCATCTGCTAC; reverse GTCTGGACCGTGATGTCCTC. SOX9 forward AACGCCGAGCTCAGCAAGA; reverse TGGTACTTGTAGTCCGGGTGGTC. GAPDH forward CCCTCAATGACCACTTTGTGAA; reverse AGGCCATGTGGACCATGAG. The real-time PCR was performed in Thermal Cycler Dice® TP800 (TakaraBio, Ohtsu, Japan) by using SYBR® Premix Ex TaqTM (TakaraBio, Ohtsu, Japan). cDNA template (5 ng) was used for real-time PCR in a final volume of 25 microliters. cDNA was amplified according to the following conditions: 95 degrees Celsius for 5 sec and 60 degrees Celsius for 30 sec at 40 amplification cycles. Fluorescene changes were monitored with SYBR Green after every cycle. A dissociation curve analysis was performed (0.5 degrees Celsius /sec increase from 60 to 95 degrees Celsius with continuous fluorescene readings) at the end of cycles to ensure that single PCR products were obtained. The amplicon size and reaction specificity were confirmed by 2.5% agarose gel electrophoresis. The results were evaluated using the Thermal Cycler Dice® Real Time System software program (TakaraBio, Ohtsu, Japan). Glyceroaldehyde-3-phosphate dehydrogenase (GAPDH) primers were used to normalize samples.

### Statistical analysis

A priori power analysis was performed. A sample size was calculated to have 70% to 83% power to test the hypothesis. All data were described as the mean and standard deviation values. The one-way analysis of variance (ANOVA) was performed with the Fischer’s PLSD test for post hoc multiple comparisons. The significance limit was set at p = 0.05. For calculation, the StatView 5.0 software (SAS Institute Inc., Cary, NC, USA) was used.

## Results

### Gross observation

**Figure 2:**
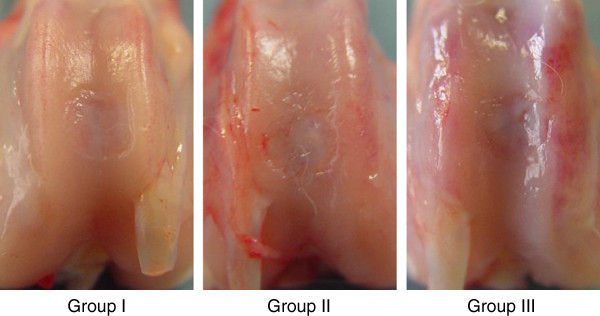
In gross observation, the defects in Groups I and II were filled with a white opaque elastic tissue, while the defects in Group III showed reddish patchy tissues.

**Figure 3:**
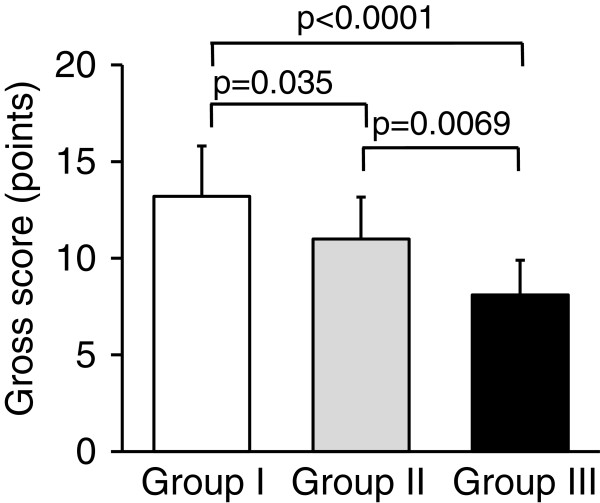
**Concerning the Wayne’s score on gross obsevation at 12 weeks, the ANOVA showed a significant difference among the 3 groups (p < 0.0001).** The post-hoc test indicated that Group I was significantly greater than Groups II and III, while Group II was significantly greater than Group III.

Postoperatively, all animals could put full weight on their limbs without restriction of motion and limping 1 week after surgery. At the death of the animals, we confirmed that there were no findings indicating infection of the knee joint, for example, swelling or purulent discharge. In gross observation, we did not observe any inflammatory findings or any pathological changes in each knee joint at 12 weeks. The defects in Groups I and II were filled with a white opaque elastic tissue, while the defects in Group III showed reddish patchy tissues (Figure 2). Concerning the Wayne’s score at 12 weeks, the ANOVA showed a significant difference among the 3 groups (p < 0.0001) (Table 1). The post-hoc test indicated that Group I was significantly greater than Groups II and III (p = 0.0350 and p < 0.0001, respectively), while Group II was significantly greater than Group III (p = 0.0069) (Figure 3).

**Table 1:** Gross appearance

	**Group I**	**Group II**	**Group III**	**P value**
**Total score**	**13.2 (2.6)**^**a, b**^	**11.0 (2.2)**^**b**^	**8.1 (1.8)**	**< 0.0001**
Sub score				
Coverage	3.7 (0.7)^a, b^	3.0 (0.5)	2.4 (0.8)	0.0009
Neocartilage color	3.1 (0.6)^b^	2.8 (0.6)^b^	2.2 (0.4)	0.0036
Defect margins	3.1 (0.6)^b^	2.8 (0.6)^b^	1.9 (0.7)	0.0009
Surface	3.3 (1.2)^a, b^	2.4 (0.7)	1.6 (0.7)	0.0008

^a^Significantly different from Group II (P < 0.05).

^b^Significantly different from Group III (P < 0.05).

### Histological and immunohistochemical observations

**Figure 4:**
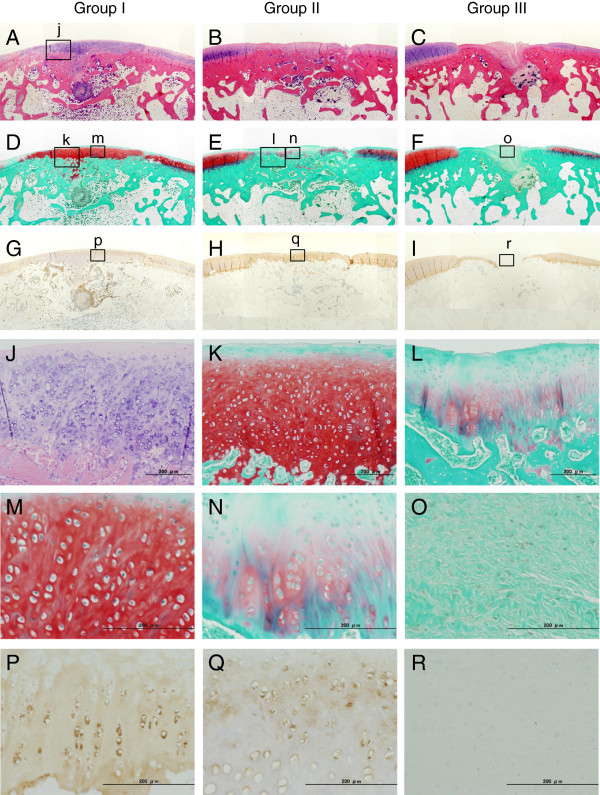
**Histological observations of the defect at 12 weeks (A-C: Hematoxylin-Eosin staining, D-F: Safranin-O staining, G-I: immuno-histological staining for type II collagen, original magnification ×2).** Group I was filled with a proteoglycan-rich tissue, in which type 2 collagen was abundantly expressed (**A**, **D**, and **G**). In high magnification histology of Group I, no cleft was observed between the regenerated tissue and the normal cartilage tissue (j: A black bar shows 200 micrometer). In the regenerated tissue, fairly large round cells rich in cytoplasm were scattered singly or as an isogenous group in a proteoglycan-rich matrix (k, l, p). In Group II, the defect was filled with thin heterogeneous tissues, including cartilage, fibrous, and bone tissues at 12 weeks (**B** and **E**). In this tissue, type 2 collagen was not uniformly expressed (**H**, q). In this tissue, cells were rather small and sparse (m, n). In Group III, the defect was filled with the fibrous and bone tissues (Figure **C** and **F**), while the type 2 collagen expression was not found in this tissue (**I**, r).

**Figure 5:**
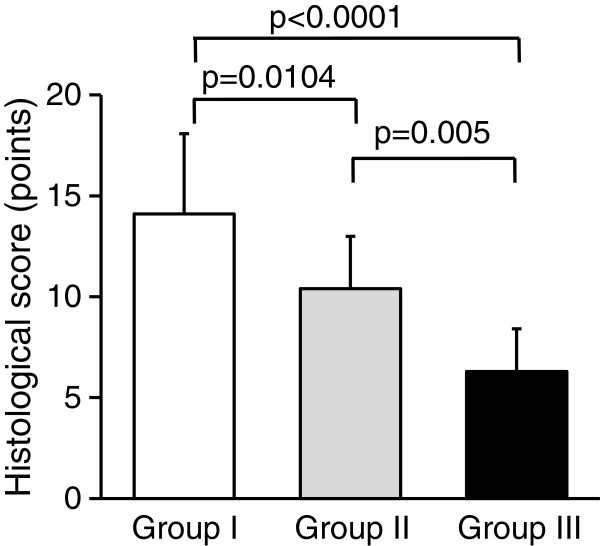
**The histological scores at 12 weeks.** The ANOVA showed a significant difference among the 3 groups (p < 0.0001). The post-hoc test indicated that Group I was significantly greater than Groups II and III, while Group II was significantly greater than Group III.

At 12 weeks, we did not observe any inflammatory findings in the synovial tissue of each knee joint. The defect in Group I was filled with a proteoglycan-rich tissue, while the width of this tissue was approximately the same as that of the normal articular cartilage (Figure 4A, D). In the proteoglycan-rich tissue, type 2 collagen was abundantly expressed (Figure 4G, p). In high magnification histology of Group I, no cleft was observed between the regenerated tissue and the normal cartilage tissue (Figure 4j). In the regenerated tissue, fairly large round cells rich in cytoplasm were scattered singly or as an isogenous group in a proteoglycan-rich matrix (Figure 4k, m). In Group II, the defect was filled with thin heterogeneous tissues, including cartilage, fibrous, and bone tissues at 12 weeks (Figure 4B, E, l, n). The width of this tissue was approximately a half of the normal cartilage. In this tissue, type 2 collagen was not uniformly expressed (Figure 4H, q). In this tissue, cells were rather small and sparse (Figure 4n). In Groups I and II, the implanted collagen was completely absorbed. In Group III, the defect was filled with the fibrous and bone tissues (Figure 4C, F, o), while the type 2 collagen expression was not found in this tissue (Figure 4I, r). Concerning the histological scores at 12 weeks, the ANOVA showed a significant difference among the 3 groups (p < 0.0001) (Table 2). The post-hoc test indicated that Group I was significantly greater than Groups II (p = 0.0104) and III (p < 0.0001), while Group II was significantly greater than Group III (p = 0.0050) (Figure 5). The cell density in the proteoglycan-rich tissue was significantly higher in Group I than in Groups II (p = 0.0001) and III (p < 0.0001), and significantly higher in Group II than in Group III (p < 0.0001) (Table 3).

**Table 2:** Histology

	**Group I**	**Group II**	**Group III**	**P value**
**Total score**	**14.1 (4.0)**^**a, b**^	**10.4 (2.6)**^**b**^	**6.3 (2.1)**	**< 0.0001**
Sub score				
Matrix	2.7 (1.3)^b^	1.9 (0.6)^b^	0.9 (0.6)	0.0006
Cell distribution	2.4 (0.5)^b^	1.9 (0.9)	1.4 (0.5)	0.0082
Surface	3.6 (0.7)^a, b^	2.6 (0.8)^b^	1.8 (0.4)	< 0.0001
Safranin at O stain	2.7 (0.9)^b^	2.1 (0.9)^b^	1.1 (0.6)	0.0006
Percent Safranin O in defect	2.7 (1.1)^a, b^	1.9 (0.9)^b^	1.1 (0.6)	0.0012

^a^Significantly different from Group II (P < 0.05).

^b^Significantly different from Group III (P < 0.05).

**Table 3:** Cell density

	**Group I**	**Group II**	**Group III**	**P value**
Cell density Per mm^2^	592.7 (130.0)^a,b^	280.7 (104.1)^b^	0 (0)	< 0.0001

^a^Significantly different from Group II (P < 0.05).

^b^Significantly different from Group III (P < 0.05).

### Real-time PCR analysis

**Figure 6:**
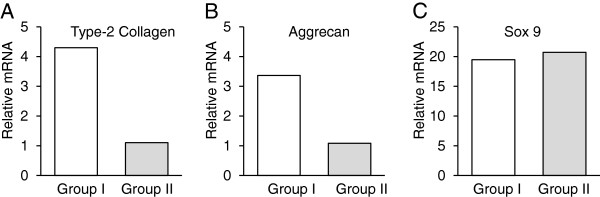
**Real-time PCR analysis on gene expression of type-2 collagen, aggrecan, and SOX9 at 12 weeks. mRNA expression of type-2 collagen (A) and aggrecan (B) of Group I was 5 times and 3 times, respectively, as much as that of Group II.** SOX9 (**C**) was highly expressed in both Groups I and II to the same degree.

The real time PCR analysis showed that gene expression of type-2 collagen and aggrecan of Group I was 5 times and 3 times, respectively, as much as that of Group II (Figure 6). SOX9 was highly expressed in both Groups I and II to the same degree.

## Discussion

The present study showed, first, that the OP1-SCS disc did not show any detrimental effects in the knee joint, when they were implanted into an osteochondral defect created in the rabbit knee. These results showed that the fish-derived collagen sponge-OP-1complex can be used as a safe implant material from the viewpoint of the immunological reaction. Secondly, this study indicated that the implanted OP1-SCS disc was completely absorbed at 12 weeks. This result was supported by our previous study that the absorption rate of the crosslinked SC sponge was significantly higher than the crosslinked porcine collagen sponge [12]. The excellent absorbability is considered to be one of advantages of this implantation device for cartilage repair. Thirdly, the quantitative comparisons in this study showed that the Wayne’s score of Group I was significantly higher than that of Groups II and III. In addition, gene expression of type-2 collagen and aggrecan of Group I was 5 times and 3 times, respectively, as much as that of Group II. The fact that SOX9 was highly expressed in both Groups I and II to the same degree may be explained by the findings that chondrocyte differentiation was induced in both the 2 groups. Thus, the present study clearly demonstrated that the implantation of the OP1-SCS disc without any cultured cells may induce spontaneous hyaline-like cartilage regeneration to greater degrees than implantation of only the salmon-derived collagen sponge disc.

There are a few differences between the salmon-derived and mammalian-derived collagens: First, the hydroxyproline content of the salmon-derived collagen is about half of that of the porcine collagen [10]. The low content of hydroxyl group in the salmon-derived collagen leads to a higher hydrophobicity of salmon-derived collagen than that of porcine collagen. Secondly, manner of integrin-mediated cell attachment to a collagen peptide is different between the salmon-derived collagen and other collagens, For example, Nagai et al. [11] showed that human umbilical vein endothelial cell attachment to the salmon-derived collagen gel depends on the alpha v beta 3 integrins as well as the alpha 2 beta 1 integrin, while the human umbilical vein endothelial cell attachment to the bovine collagen gel depends on the alpha 2 beta 1 integrin. Integrins not only mediate cell adhesion to the extracellular matrix but also activate intracellular signaling pathways [20].

It was noted that implantation of only the salmon-derived crosslinked collagen sponge disc had a mild but significant effect that induced hyaline-like cartilage regeneration in an osteochondral defect at 12 weeks in comparison with the no treatment control. We can speculate possible reasons to explain this phenomenon. First, in the patellofemoral joint, immediate motion and loading results in high shear forces, preventing the formation of an blood clot and might also be the reason for poor cartilage regeneration. Therefore, first, the function of this cell-free scaffold is to provide a temporary structure while cells seeded within the biodegradable matrix synthesize hyaline-like cartilage tissue. Secondly, the salmon-derived collagen appears to have higher biological functions than the mammalian-derived collagen [12]. For example, it is reported that human periodontal ligament fibroblasts showed better growth and higher alkaline phosphatase activity in culture on the salmon-derived collagen than in the culture with the porcine collagen [10]. Thirdly, there is a possibility that an increase of the degree of crosslinking in the the salmon-derived collagen plays an important role in the induction effect of hyaline-like cartilage regeneration [12]. There are a few reports that the crosslinking of collagen molecules enhanced the DNA synthesis of epidermal cells [20] and the proliferation of endothelial cells [21]. In the salmon-derived atelocollagen disc used in the present study, the degree of reduction of the free-amino residues was about 20% [9]. The reduction of free-amino residues changes the chemical properties of the salmon-derived atelocollagen [9]. In addition, crosslinking changes the mechanical properties of the collagen sponges. It is known that the mechanical properties of the scaffold affect the cellular functions and morphologies [22]. Thus, we consider that these mechanisms may enhance the ability of the salmon-derived collagen, which induces hyaline-like cartilage regeneration. However, further biological and biomechanical studies are needed to clarify how the above-described differences in the collagen sponges affect the cartilage regeneration.

We considered the mechanism of the effect of OP-1. Previous *in vitro* studies showed that OP-1 up-regulates chondrocyte metabolism and enhances protein and proteoglycan synthesis in chondrocytes [23-27], Also recent studies have shown that OP-1 stimulates cartilage-specific extracellular proteins, such as type II and VI collagens, aggrecan, decorin, fibronectin, hyaluronan, and so on, in chondrocytes [16,28]. Furthermore, OP-1 has anti-catabolic activities for chondrocytes. Namely, OP-1 effectively counteracts chondrocyte catabolism induced by various catabolic mediators, such as proinflammatory cytokines and fragments of cartilage matrix proteins [29,30]. OP-1 also modulates the expression of various catabolic mediators [31]. Therefore, there is a possibility that the OP-1 might affect the chondrocytes, which were differentiated from mesenchymal stem cells by existence of the salmon-derived crosslinked collagen sponge, as shown in our previous study [12]. In addition, several *in vivo* studies reported that OP-1 itself promotes chondrogenic differentiation and matrix formation [32-34]. Therefore, there is also a possibility that the applied OP-1 additionally promoted chondrogenic differentiation of the mesenchymal stem cells which migrated into the sponge, and enhanced synthesis of cartilage-specific extracellular proteins and proteoglycans.

There are some limitations in this study. The first limitation is that we did not perform long-term observation of the regenerated cartilage. A long-term evaluation study is needed to be performed immediately after this study. The second limitation is that we did not perform statistical analysis concerning the gene expression of type-2 collagen, aggrecan, and SOX9 for the insufficient number of specimen. In addition, we did not evaluate the gene expression of type-1, and −10 collagen. Thirdly, we did not perform a biochemical and biomechanical analyses of the regenerated cartilage. Fourthly, we did not perform both in vitro and in vivo studies on the release/production of OP-1 from the sponge. However, Hayashi et al. [35] demonstrated that the cartilage was obtained from rabbit knee joints at 1 hour and 1, 2, 4, and 7 days after intra-articular injection of 500 ng OP-1. 1 hour after BMP-7 injection, BMP-7 was detected at high levels in cartilage; the level decreased by 60% 1 day after injection. BMP-7 levels gradually decreased over time, and 7 days after the last injection BMP-7 concentration in treated knees was still higher than that in control knees. Therefore, we speculated that in the present study there was the similar condition on the elution of the factors from the sponge. Fifthly, we could not determine the mechanism of the in vivo cartilage regeneration induced by the the OP1-SCS disc in the present experiment. The sixth limitation is that we implanted the sponge into the patellofemoral joint because this joint is commonly used in animal studies to develop a new treatment for a cartilage defect. Therefore, we cannot refer to the effect of implantation of the artificial gel cartilage into the tibiofemoral joint. At the present time, however, it is important to increase the database concerning *in vivo* effects of cell-free devices for cartilage regeneration. From this viewpoint, we believe that this study is of value to propose a therapeutic concept using a novel salmon-derived collagen device.

As to clinical relevance, this study implied that the OP1-SCS disc is a promising cell-free device for cartilage repair. This disc has great advantages including no risk of zoonosis transmission, a quickly absorbable device, a large source for commercial production with a low cost. In addition, it is known that an application of OP-1 has been clinically tried to treat nonunion of the fracture and massive bone and to promote spine fusion in the field of orthopaedic surgery [36,37]. Therefore, the safty of OP-1 has been established for clinical use. We believe that the OP1-SCS disc is of value to be studied more as a potential therapeutic device for cartilage repair in the near future. For example, further long-term studies using large animals are needed to establish the efficacy (proof of concept) of this device.

## Conclusion

The present study showed that the histological score of Group I was significantly higher than that of Groups II and III. In addition, gene expression of type-2 collagen and aggrecan of Group I was greater than that of Group II. Thus, the present study clearly demonstrated that the implantation of the OP1-SCS disc without any cultured cells may induce spontaneous hyaline-like cartilage regeneration to greater degrees than implantation of only the salmon-derived collagen sponge disc.

## Competing interests

We have no financial or non- financial competing interests. We do not hold or are not currently applying for any patents relating to the content of the manuscript.

## Authors’ contributions

HM performed animal experiments, and the histological and PCR analyses. EK, KY, and HI designed the study, participated in the study, and drafted the manuscript. YK and NK participated in designing the study and instructed animal experiments. NN created the salmon-derived atelocollagen sponge material. All authors read and approved the final manuscript.

## Pre-publication history

The pre-publication history for this paper can be accessed here:

http://www.biomedcentral.com/1471-2474/14/174/prepub

## References

[B1] Brittberg M Lindahl A Nilsson A Ohlsson C Isaksson O Peterson L Treatment of deep cartilage defects in the knee with autologous chondrocyte transplantation New Engl J Med 1994 331 889 895 10.1056/NEJM199410063311401 8078550

[B2] Peterson L Minas T Brittberg M Lindahl A Treatment of osteochondritis dissecans of the knee with autologous chondrocyte transplantation: results at two to ten years J Bone Joint Surg Am 2003 85 17 24 10.1302/0301-620X.85B1.13948 12721341

[B3] Ochi M Adachi N Nobuto H Yanada S Ito Y Agung M Articular cartilage repair using tissue engineering technique–novel approach with minimally invasive procedure Artif Organs 2004 28 28 32 10.1111/j.1525-1594.2004.07317.x 14720285

[B4] Knutsen G Drogset JO Engebretsen L Grøntvedt T Isaksen V Ludvigsen TC Roberts S Solheim E Strand T Johansen O A randomized trial comparing autologous chondrocyte implantation with microfracture. Findings at five years J Bone Joint Surg Am 2007 89 2105 2112 10.2106/JBJS.G.00003 17908884

[B5] Swatschek D Schatton W Kellermann J Müller WE Kreuter J Marine sponge collagen: isolation, characterization and effects on the skin parameters surface-pH, moisture and sebum Eur J Pharm Biopharm 2002 53 107 113 10.1016/S0939-6411(01)00192-8 11777758

[B6] Yunoki S Suzuki T Takai M Stabilization of low denaturation temperature collagen from fish by physical cross-linking methods J Biosci Bioeng 2003 96 575 577 10.1016/S1389-1723(04)70152-8 16233576

[B7] Burjanadze TV Thermodynamic substantiation of water-bridged collagen structure Biopolymers 1992 32 941 949 10.1002/bip.360320805 1420978

[B8] Nagai N Yunoki S Suzuki T Sakata M Tajima K Munekata M Application of crosslinked salmon atelocollagen to the scaffold of human periodontal ligament cells J Biosci Bioeng 2004 97 389 394 16233648 10.1016/S1389-1723(04)70224-8

[B9] Yunoki S Nagai N Suzuki T Munekata M Novel biomaterial from reinforced salmon collagen gel prepared by fibril formation and cross-linking J Biosci Bioeng 2004 98 40 47 16233664 10.1016/S1389-1723(04)70240-6

[B10] Nagai N Mori K Saoh Y Takahashi N Yunoki S Tajima K Munekata M In vitro growth and differentiated activities of human periodontal ligament fibroblasts cultured on salmon collagen gel J Biomed Mater Res A 2007 82 395 402 17295232 10.1002/jbm.a.31110

[B11] Nagai N Mori K Munekata M Biological properties of crosslinked salmon collagen fibrillar gel as a scaffold for human umbilical vein endothelial cells J Biomater Appl 2008 23 275 287 10.1177/0885328208092109 18697879

[B12] Kawaguchi Y Kondo E Kitamura N Arakaki K Tanaka Y Munekata M Nagai N Yasuda K In vivo effects of isolated implantation of salmon-derived crosslinked atelocollagen sponge into an osteochondral defect J Mater Sci Mater Med 2011 22 397 404 10.1007/s10856-010-4215-1 21259035

[B13] Sakou T Bone morphogenetic proteins: from basic studies to clinical approaches Bone 1998 22 591 603 10.1016/S8756-3282(98)00053-2 9626397

[B14] Loeser RF Pacione CA Chubinskaya S The combination of insulin-like growth factor-1 and osteogenic protein-1 promotes increased survival of and matrix synthesis by normal and osteoarthritic human articular chondrocytes Arthritis Rheum 2003 48 2188 2196 10.1002/art.11209 12905472

[B15] Chubinskaya S Merrihew C Cs-Szabo G Mollenhauer J McCartney J Rueger DL Kuettner KE Human articular chondrocytes express osteogenic protein-1 J Histochem Cytochem 2000 48 239 250 10.1177/002215540004800209 10639490

[B16] Chubinskaya S Hakimiyan A Pacione C Yanke A Rappoport L Aigner T Rueger D Loeser RF Synergistic effect of IGF-1 and OP-1 on matrix formation by normal and OA chondrocytes cultured in alginate beads Osteoarthritis Cartilage 2007 15 421 430 10.1016/j.joca.2006.10.004 17126570 PMC1894688

[B17] Chubinskaya S Kawakami M Rappoport L Matsumoto T Migita N Rueger DC Anti-catabolic effect of OP-1 in chronically compressed intervertebral discs J Ortho Res 2007 25 517 530 10.1002/jor.20339 17205567

[B18] Hayashi R Kondo E Tohyama H Saito T Yasuda K In vivo local administration of osteogenic protein-1 increases structural properties of the overstretched anterior cruciate ligament with partial midsubstance laceration. A biomechanical study in rabbits J Bone Joint Surg Br 2008 90 1392 1400 18827254 10.1302/0301-620X.90B10.20924

[B19] Wayne JS McDowell CL Shields KJ Tuan RS In vivo response of polylactic acid-alginate scaffolds and bone marrow-derived cells for cartilage tissue engineering Tissue Eng 2005 11 953 963 10.1089/ten.2005.11.953 15998234

[B20] Nishikawa A Taira T Yoshizato K In vitro maturation of collagen fibrils modulates spreading, DNA synthesis, and collagenolysis of epidermal cells and fibroblasts Exp Cell Res 1987 171 164 177 10.1016/0014-4827(87)90259-X 3040448

[B21] Kuzuya M Satake S Ai S Asai T Kanda S Ramos MA Miura H Ueda M Iguchi A Inhibition of angiogenesis on glycated collagen lattices Diabetologia 1998 41 491 499 10.1007/s001250050937 9628264

[B22] Semler EJ Ranucci CS Moghe PV Mechanochemical manipulation of hepatocyte aggregation can selectively induce or repress liver-specific function Biotechnol Bioeng 2000 69 359 369 10.1002/1097-0290(20000820)69:4<359::AID-BIT2>3.0.CO;2-Q 10862674

[B23] Flechtenmacher J Huch K Thonar EJ-MA Mollenhauer JA Davies SR Schmid TM Puhl W Sampath TK Aydelotte MB Kuettner KE Recombinant human osteogenic protein 1 is a potent stimulator of the synthesis of cartilage proteoglycans and collagens by human articular chondrocytes Arthritis Rheum 1996 39 1896 1904 10.1002/art.1780391117 8912513

[B24] Lietman S Yanagishita M Sampath TK Reddi AH Stimulation of proteoglycan synthesis in explants of porcine articular cartilage by recombinant osteogenic protein-1 (bone morphogenetic protein-7) J Bone Joint Surg Am 1997 79 1132 1137 9278071 10.2106/00004623-199708000-00003

[B25] Nishida Y Knudson CB Eger W Kuettner KE Knudson W Osteogenic protein-1 stimulates cell-associated matrix assembly by normal human articular chondrocytes: upregulation of hyaluronan synthase, CD 44 and aggrecan Arthritis Rheum 2000 43 206 214 10.1002/1529-0131(200001)43:1<206::AID-ANR25>3.0.CO;2-1 10643717

[B26] Chubinskaya S Kumar B Merrihew C Heretis K Rueger D Kuettner KE Age-related changes in cartilage endogenous OP-1 Biochimica Biophysica Acta Mol Basis Dis 2002 1588 126 134 10.1016/S0925-4439(02)00158-8 12385776

[B27] Fan Z Chubinskaya S Rueger DC Bau B Haag J Aigner T Regulation of anabolic and catabolic gene expression in normal and osteoarthritic adult human articular chondrocytes by OP-1 (BMP-7) J Clin Exper Rheum 2004 22 103 106 15005012

[B28] Loeser R Chubinskaya S Pacione C Im H-J Basic fibroblast growth factor inhibits the anabolic activity of insulinlike growth factor-1 and osteogenic protein-1 in adult human articular chondrocytes Arthritis Rheum 2005 52 3910 3917 10.1002/art.21472 16320338 PMC1482464

[B29] Huch K Wilbrink B Flechtenmacher J Koepp HE Aydelotte MB Sampath TK Kuettner KE Mollenhauer JA Thonar EJMA Effects of recombinant human osteogenic protein 1 on the production of proteoglycan, prostaglandin E2, and interleukin-1 receptor antagonist by human articular chondrocytes cultured in the presence of interleukin-1beta Arthritis Rheum 1997 40 2157 2161 10.1002/art.1780401209 9416852

[B30] Koepp HE Sampath KT Kuettner KE Homandberg GA Osteogenic protein-1 (OP-1) blocks cartilage damage caused by fibronectin fragments and promotes repair by enhancing proteoglycan synthesis Inflamm Res 1999 47 1 6 10.1007/s000110050446 10344470

[B31] Im HJ Pacione C Chubinskaya S VanWijnen AJ Sun Y Loeser RF Inhibitory effects of insulin-like growth factor-1 and osteogenic protein-1 on fibronectin fragment- and interleukin-1beta-stimulated matrix metalloproteinase-13 expression in human chondrocytes J Biol Chem 2003 278 25386 25394 10.1074/jbc.M302048200 12734180 PMC2895259

[B32] Louwerse RT Iheyligers IC Klein-Nulend J Sugiihara S van Kampen GPJ Semeins CM Goei SW de Koning MHMT Wuisman PIJM Burger EH Use of recombinant human osteogenic protein-1 for the repair of subchondral defects in articular cartilage in goats J Biomed Mater Res 2000 49 506 516 10.1002/(SICI)1097-4636(20000315)49:4<506::AID-JBM9>3.0.CO;2-A 10602084

[B33] Jelic M Pecina M Haspl M Kos J Taylor K Maticic D McCartney J Yin S Rueger D Vukicevic S Regeneration of articular cartilage chondral defects by osteogenic protein-1 (bone morphogenetic protein-7) in sheep Growth Fact 2001 19 101 113 10.3109/08977190109001079 11769970

[B34] Cook SD Preclinical and clinical evaluation of osteogenic protein-1 (BMP-7) in bony sites Orthopedics 1999 22 669 671 10418861

[B35] Hayashi M Muneta T Ju YJ Mochizuki T Sekiya I Weekly intra-articular injections of bone morphogenetic protein-7 inhibits osteoarthritis progression Arthritis Res Ther 2008 10 5 R118 10.1186/ar2521 18826579 PMC2592805

[B36] Friedlaender GE Perry CR Cole JD Cook SD Cierny G Muschler GF Zych GA Calhoun JH LaForte AJ Yin S Osteogenic protein-1 (bone morphogenetic protein-7) in the treatment of tibial nonunions J Bone Joint Surg Am 2001 83 151 158 11314793 PMC1425155

[B37] Kanayama M Hashimoto T Shigenobu K Yamane S Bauer TW Togawa D A prospective randomized study of posterolateral lumbar fusion using osteogenic protein-1 (OP-1) versus local autograft with ceramic bone substitute: emphasis of surgical exploration and histologic assessment Spine 2006 31 1067 1074 10.1097/01.brs.0000216444.01888.21 16648739

